# Telomere length is longer following diapause in two solitary bee species

**DOI:** 10.1038/s41598-024-61613-2

**Published:** 2024-05-16

**Authors:** Courtney C. Grula, Joshua D. Rinehart, Angelo Anacleto, Jeffrey D. Kittilson, Britt J. Heidinger, Kendra J. Greenlee, Joseph P. Rinehart, Julia H. Bowsher

**Affiliations:** 1 Insect Genetics and Biochemistry Edward T. Schafer Research Center, U.S. Department of Agriculture/Agricultural Research Center, 1616 Albrecht Boulevard, Fargo, ND 58102 USA; 2 Department of Biological Sciences, North Dakota State University, 1340 Bolley Drive, 218 Stevens Hall, Fargo, ND 58102 USA; 3 Department of Molecular and Integrative Physiology, University of Michigan Medical School, 1137 E. Catherine St., Ann Arbor, MI 48109 USA

**Keywords:** Physiology, Ageing, Senescence

## Abstract

The mechanisms that underlie senescence are not well understood in insects. Telomeres are conserved repetitive sequences at chromosome ends that protect DNA during replication. In many vertebrates, telomeres shorten during cell division and in response to stress and are often used as a cellular marker of senescence. However, little is known about telomere dynamics across the lifespan in invertebrates. We measured telomere length in larvae, prepupae, pupae, and adults of two species of solitary bees, *Osmia lignaria* and *Megachile rotundata*. Contrary to our predictions, telomere length was longer in later developmental stages in both *O. lignaria* and *M. rotundata.* Longer telomeres occurred after emergence from diapause, which is a physiological state with increased tolerance to stress. In *O. lignaria*, telomeres were longer in adults when they emerged following diapause. In *M. rotundata*, telomeres were longer in the pupal stage and subsequent adult stage, which occurs after prepupal diapause. In both species, telomere length did not change during the 8 months of diapause. Telomere length did not differ by mass similarly across species or sex. We also did not see a difference in telomere length after adult *O. lignaria* were exposed to a nutritional stress, nor did length change during their adult lifespan. Taken together, these results suggest that telomere dynamics in solitary bees differ from what is commonly reported in vertebrates and suggest that insect diapause may influence telomere dynamics.

## Introduction

Stressful environmental conditions, especially those experienced during early life, often have negative long-term consequences for fitness^1^. One mechanism that may be an important biomarker of early life conditions is telomere dynamics^2,3^. Telomeres are highly conserved, repetitive sections of non-coding DNA at chromosome ends. Telomeres protect coding DNA from erosion during replication and enhance genome integrity. Telomeres shorten during cell division and are expected to play an important role in cellular and organismal senescence^4^. In support of this, declining telomere length with age has been detected in a variety of vertebrates^5^, including humans^6–8^ birds^9–13^, and fish^14,15^. In addition, telomere length is often positively correlated with longevity in many organisms^16–22^. Telomere length is often heritable^23^ and average telomere length can differ between populations^24^. Exposure to a range of environmental stressors can accelerate telomere loss^25^. Nutritional stress, for example, may trigger physiological responses that impact cellular maintenance and repair processes, including telomere length regulation^26^. The positive relationship between telomeres and lifespan starts early in life^16,27,28^. In longitudinal studies, telomere loss is often greater during juvenile development^16^, and exposure to stress during early life often increases telomere loss^29,30^.

Telomere length and dynamics are variable across species and respond differently to life history traits^31,32^. While much is known about telomere dynamics in vertebrates, a significant gap exists in our understanding of how telomeres function in invertebrates, particularly in insects. In general, invertebrates show a pattern of telomere shortening or maintaining length over time;^33^ insects specifically maintain telomere throughout the lifespan^34–37^. Although, telomere lengthening has also been observed in invertebrates^38^. In social insects (honeybees and bumblebees), reproductive individuals (queens) have significantly longer lifespans than workers, but telomere length does not differ between castes^35–37^. Telomerase is up-regulated in social bee queens, maintaining telomere length over the longer lifespan^35–37^. However, most bee species are solitary and have annual lifespans and life cycles similar to other insects^39,40^, and it is not known whether telomere length is maintained throughout the course of a year and in each life stage.

Temperate insects have annual life cycles characterized by an overwintering stage known as diapause. Diapause is a physiological state of decreased metabolism and increased tolerance to cold and stress^41^. In some species, diapause is facultative with diapausing individuals having lifespans that are many months longer than that of non-diapausing individuals^42^. The physiological mechanisms down-regulated during diapause share similarities with laboratory extensions of lifespan^43^. Like diapause, vertebrate hibernation involves a decrease in metabolic rate^44^. In some hibernating species, telomere shortening slows down or even stops during the period of metabolic suppression^45–48^. There is some evidence that diapause can slow senescence^49–51^, but that has not been linked to telomere dynamics. Understanding how diapause influences telomere dynamics may give insights about how overwintering survival strategies benefit insects.

We measured telomere length in two solitary bee species, *Megachile rotundata* (F.) and *Osmia lignaria* (Say), at multiple stages across the lifecycle. Telomere dynamics have not been measured to date in solitary bees, which make up the majority of the species in the order Hymenoptera^39,40^. Results from studying telomeres in *M. rotundata* and *O. lignaria* may be more generalizable to other insects and bees because they have diapause strategies that are more similar to the bees in our study. Our first aim was to determine whether telomere length changes across the lifespan. We predicted that telomere length would be shorter in later developmental stages because declining telomere length with age has been documented in many species. We also examined other factors that have previously been shown to impact telomere length including sex, body size, and nutritional status. We predicted telomere length would be shorter in larger individuals. We also predicted that telomere length would be longer in females because females in both species typically live longer than males which may be reflected in their telomere dynamics. To determine how stress impacts telomere dynamics, we measured telomere length after exposure to a nutritional stress. We predicted that telomeres would shorten after stress exposure and this may be amplified in older individuals. We were also interested in measuring telomere length during diapause to determine if telomere dynamics change during this period of arrest. We predicted that time spent in diapause would decrease telomere loss. Contrary to our predictions, telomere length was maintained under most conditions. However, we observed that telomeres were longer after bees emerged from diapause.

## Methods

### Study system

**Figure 1:**
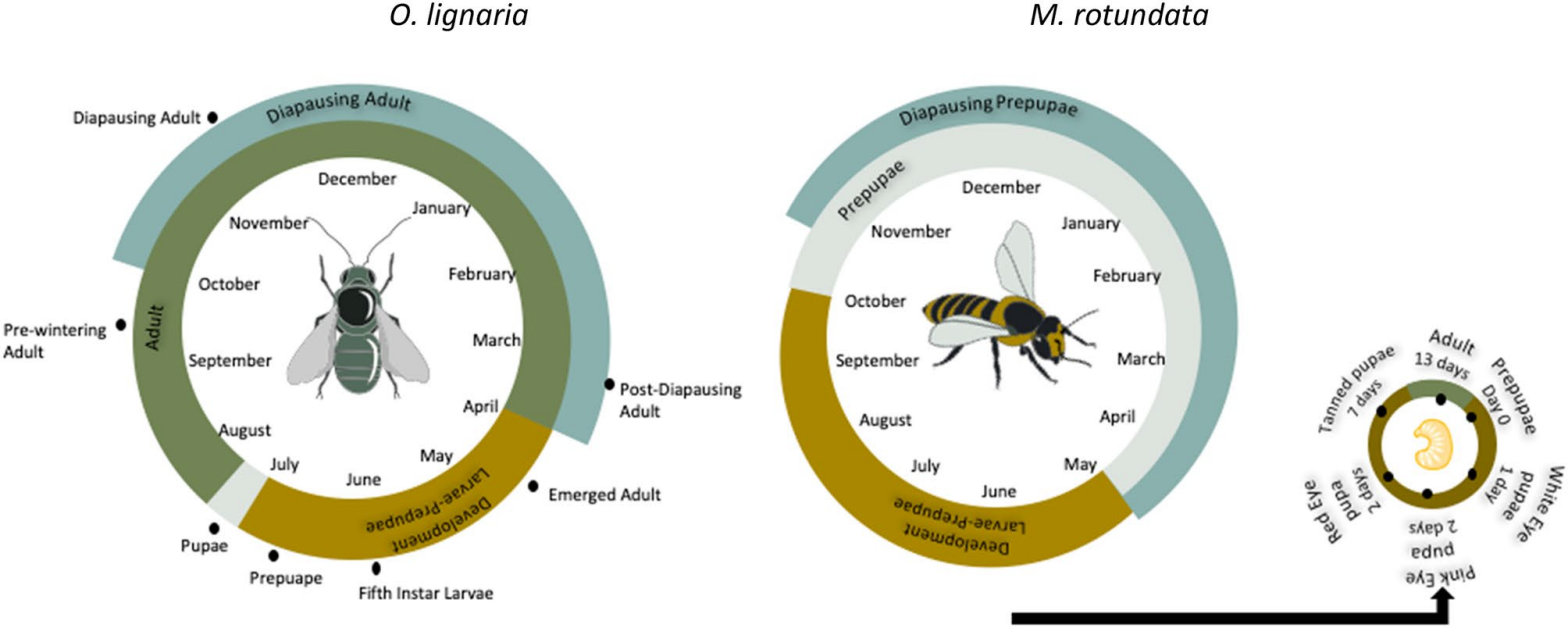
The life cycle of *O. lignaria* (left) and *M. rotundata* (right). *O. lignaria* spends most of its lifespan as an adult, majority of that time occurs during the overwintering stage. *M. rotundata* overwinter as prepapae and spend very little of their lifespan as adults. For *M. rotundata*, the entire active season for adults is denoted, with individual adults living for only a few weeks. Colors denote life stages. Black dots denote sampling points.

The solitary bees *O. lignaria* and *M. rotundata* differ in which life stage they overwinter, as well as the amount of time spent as adults (Fig. 1). Solitary bees differ from social bees in that each female builds her own nest and provisions her own offspring, and also has differing diapause strategies.

*O. lignaria* has an obligate diapause stage as an adult^52,53^. Pupal development in *O. lignaria* occurs before overwintering and they begin winter diapause after they have become adults*. O. lignaria* bees are in the adult stage for around eight months and emerge in early spring. In contrast, *M. rotundata* overwinters in the prepupal stage, which begins during the late summer^54^. *M. rotundata* are facultative diapausers, and some individuals will skip diapause and emerge the same summer, while others will enter diapause as a prepupae, emerging the following summer. Only diapausing bees were used in this study. Focusing on diapausers allowed for generalized comparisons between *O. lignaria*, and *M. rotundata*, because *O. lignaria* are obligate diapausers. When temperatures increase in the spring, *M. rotundata* pupates and goes through the following pupal stages: white eye, pink eye, red eye, and tanning pupae. The different pupal stage can be visually identified by the color of the eyes which correspond to their developmental timing^55,56^. Adults emerge mid-summer.

### *Megachile rotundata* rearing and sample collection

Prepupal bees were sourced from JWM Leafcutters, Inc. of Nampa, ID, US (March 2020). Bees were taken from 6 °C^54^ storage, removed from their brood cells, overwintering prepupae were randomly chosen and immediately flash frozen on liquid nitrogen and stored. The rest of the bees were placed in 48 well plate in a 29 °C environmental chamber (Darwin Chambers, St. Louis, MO) to initiate development. Bees were visually monitored and randomly sampled at the following stages: overwintering prepupae (date collected 3/9/20 n = 28), white eye pupae (date collected 6/13/20; n = 28), pink eye pupae (date collected 6/15/20; n = 28), red eye pupae (date collected 6/15/20; n = 25), tanning pupae (date collected 6/19/20; n = 18), and newly emerged adult (date collected 6/26/20; n = 28). Bees were flash frozen on liquid nitrogen and stored in − 80 °C.

### *Osmia lignaria* rearing and sample collection

Freshly capped *O. lignaria* nests containing larval bees, from Woodenville, WA (2018), and CA (2021) were kept in an environmental chamber at 25 °C and 75% humidity and allowed to develop. Developmental stages were monitored by the Faxitron MX-20 cabinet X-ray with Scan-X scanner (Faxitron Bioptics LLC, Tucson, AZ). Bees from both years were randomly sampled at the following stages: fifth instar larvae (date collected May 25; n = 29), prepupae (date collected June 25; n = 32), pupae (date collected July 9; n = 38), pre-wintering adults (date collected September 19; n = 42), diapausing adults (date collected November 28; n = 43), post-diapausing adults (date collected March 12; n = 43), and emerged adults (date collected April 19; n = 45). Adult developmental timepoints were determined by developmental date. Pre-wintering adults were not exposed to cold temperatures. Bees were put into overwintering using a ramp-down temperature regime (Day 1, 22 °C; Day10, 18 °C; Day 20, 4 °C). Bees were kept at 4 °C for the duration of overwintering^57^. To initiate emergence from overwintering, temperatures were increased to 25 °C and adults that emerged were collected. Bees were flash frozen on liquid nitrogen and stored in − 80 °C.

### *Osmia lignaria* post-emergence adult sample collection

100 overwintering adult bees from Woodenville, WA were shipped to Fargo, ND. Upon arrival, a random subsample of adult bees were immediately frozen at − 80 °C for the first timepoint (day 1). The rest of the adult bees were stored in an environmental chamber at 25 °C in separate 15 ml conical tubes and fed a 1:1 sucrose solution. Bees were checked daily for survival. Bees were randomly chosen to be sampled on day 1 (n = 20), day 5 (n = 20), day 15 (n = 32), and flash frozen on liquid nitrogen and stored in the − 80 °C freezer. These timepoints were chosen because they were representative of survival in captivity. Wild Megachilid bees have a lifespan of around 30 days^58^. When in captivity, survival declines steeply around day 15^59,60^. In order to have a large sample size for telomere measurement, the last day of sampling was day 15.

### *Osmia lignaria* post-emergence adult nutritional stress

We measured telomere length in adult *O. lignaria* with the addition of a nutritional stress treatment. Bees from Logan, UT were shipped to Fargo, ND. Upon arrival, a random subset of adult bees was immediately flash frozen on liquid nitrogen (day 0, fed n = 15 starved n = 15) then stored in − 80 °C. The remaining bees were randomly placed into feeding treatments 24 h after arrival. The feeding treatments included a food removal treatment (starved) and a continuously fed treatment (fed). Starved bees were given only water for 24 h, then given a 1:1 sucrose solution for the remainder of the experimental time period. Fed bees were continuously given a 1:1 sucrose solution. Bees were stored in an incubator at 25 °C in separate 15 ml conical tubes. Bees were checked daily for survival. Bees were sampled at day 1 (fed n = 30; starved n = 25) and day 15 (fed n = 31; starved n = 23) and were then frozen on liquid nitrogen and stored in − 80 °C. These timepoints were chosen to correspond with the previous experiment.

### Telomere measurement

We have followed methods from Cawthon 2002^61^ for telomere measurement by quantitative PCR (qPCR) and have adapted this method for use in bees. We reported our telomere methods and results according to the best practices established by the Telomere Research Network^62^. Relative telomere lengths were analyzed from DNA extracted from the thorax (adult) or partial bee (larval and pupal stages) using a Nucelospin Insect DNA Extraction Kit (Macherey–Nagel, Allentown, PA). Only partial bees were used to fit the weight requirement of the kit. Quantity of DNA was measured with a NanoDrop 1000 spectrophotometer (ThermoFisher Scientific), and samples were not used if the 260/280 ratio was below 1.8. qPCR was performed on an Mx3000P qPCR system (Agilent, Santa Clara, CA) to determine relative telomere length, using the T/S ratio^61^. The T/S ratio describes the telomere signal (T) relative to a single control gene (S), which was Glyceraldehyde-3-phosphate dehydrogenase (GAPDH) in our study. GAPDH is commonly used as a control gene for telomere length^16,30^. We confirmed GAPDH is single copy in both species by BLAST to the genomes (*Megachile rotundata*, BioProject accession: PRJNA66515; *Osmia lignaria*, BioProject accession: PRJNA553797). The same telomere primers were used to amplify the telomere sequence for both *M. rotundata* and *O. lignaria* (Table 1)*.* Bee species share the conserved (TTAGG)_n_ telomere sequence^63–65^. Species-specific GAPDH primers were used (Table 1) (concentration in reaction mixture 200 nM). The telomere and GAPDH reactions were run on separate plates because PCR protocols differed. The number of PCR cycles required to accumulate a fluorescent signal to cross a threshold (0.5) was measured. The cycling parameters for the plates containing GAPDH primers was one cycle for 10 min at 95 °C, 35 cycles for 20 s at 95 °C, 39 s at 59 °C and 30 s at 72 °C, and one cycle of dissociation curve (melt curve). Our qPCR program for the plates containing telomere primers was 1 cycle for 10 min at 95 °C, 35 cycles for 20 s at 95 °C, 39 s at 58 °C, and 30 s at 72 °C and 1 cycle of dissociation for 1 min at 95 °C, 30 s at each 55 °C to 95 °C. All reactions used 20 ng of DNA in a final volume of 25 μl containing 12.5 µl of SYBR green Master Mix (PerfeCTa SYBR Green SuperMix Low ROX, Quantabio Beverly, Massachusetts) 0.25 µl forward and reverse primer, 6 µl water and 6 µl of DNA sample. A negative control of water was run on each plate. Each plate also included a non-treatment control. All samples were run in duplicate, and the standard curve was run in triplicate. We used a pool of individuals to make a standard curve that was the same across all plates. There were 5 points on the standard curve of 40, 20, 10, 5, 2.5 ng which were produced by serially diluting a reference sample. The average efficiency of the telomere plates was 103.8%. The average efficiency of the GAPDH plates was 90.7%

**Table 1:** Primer sequences.

Primer name	Primer sequence
Telo1	5′-CGG TTT GTT TGG TTT GGT TTG GTT TGG TTT GGT T-3′
Telo2	5′-GGC TTG CCT TAC CTT ACC TTA CCT TAC CTT ACC T-3′
GAPDH-F (*M. rotundata*)	5′-GACGTAGTGTCTTCCGACTTTAT -3′
GAPDH-R (*M. rotundata*)	5′-CAATCACGCGGCTAGAGTAA-3′
GAPDH-F (*O. lignaria*)	5′-GGCCAATGTCGGGAGATAAA-3′
GAPDH-R (*O. lignaria*)	5′-GAGACTCTGCTTCGCTTTCA-3′

The T/S ratio is calculated relative to a reference sample in each experiment. Reference samples in our studies were a pool of multiple individuals and used to calculate the inter-plate variation of T/S ratios across the multiple plates run for each experiment. We were able to compare telomere length between years in *O. lignaria* across developmental stages because the same reference sample was used for measuring *O. lignaria* telomeres. For the rest of the experiments in this study multiple different reference samples were used, therefore telomere length cannot be compared between all experiments.

Average duplicate values were used to calculate the T/S ratios for each sample relative to the reference sample according to the formula: 2ΔΔCt, where ΔΔCt = (Ct_telomere_−Ct_GAPDH_) reference−(Ct_telomere_−Ct_GAPDH_) sample. Repeatability of the T/S ratio was calculated by running 27 (*O. lignaria*) and 28 (*M. rotundata*) individuals from the study in plates, which had the samples randomly distributed. The intraclass correlation coefficient (ICC two-way, single measurement, absolute agreement, random effects model) was *M. rotundata* ICC = 0.86; p < 0.001, 95% confidence interval lower bound 0.86 and upper bound 0.86 *O. lignaria* ICC = 0.90 p < 0.001, 95% confidence interval lower bound 0.90 and upper bound 0.90).

### Statistical analysis

We measured telomere length in two solitary bee species to determine how telomere dynamics changed over time, as well as in response to mass, sex, and nutritional stress. All statistical analyses were performed using R statistical software (version 3.6.1, Base R package) and were graphed using the package *ggplot2*^66^. We tested for outliers and the goodness of fit for our model using the Dharma package in R^67^. We used a multiple comparisons linear model test. To control for variation among plates, qPCR plate number was included as fixed effect in all models^68^. When analyzing the effect of different developmental timepoints on telomere length in *M. rotundata*, telomere length was included as a dependent variable in the model, and mass and the interaction of mass and treatment were fixed effects. The model for determining the effect of developmental timepoints on telomere length in *O. lignaria* included telomere length as the dependent variable and developmental stage and year were fixed effects. Sex and mass, and their interactions were included as independent variables in separate models because sex and mass were not measured for all individuals. This is because sex cannot be determined before the adult stage. The model for determining the effect of adult age on telomere length in *O. lignaria* included telomere length as the dependent variable, and adult age, sex, and mass, the interaction of mass and age as well as the interaction of sex and age as fixed effects. The model for determining the effect of nutritional stress on telomere length in adult *O. lignaria* included telomere length as the dependent variable, nutritional status, adult age, sex, and mass, and the interaction of feeding treatment and age were fixed effects in the model. The interaction of developmental stage and mass was included in the model. Mass was mean-centered by life stage in all models to ensure we were detecting the effect of mass within each life stage. The *multcomp* package^69^ was used for Tukey post-hoc comparisons.

## Results

### Megachile rotundata development

**Figure 2:**
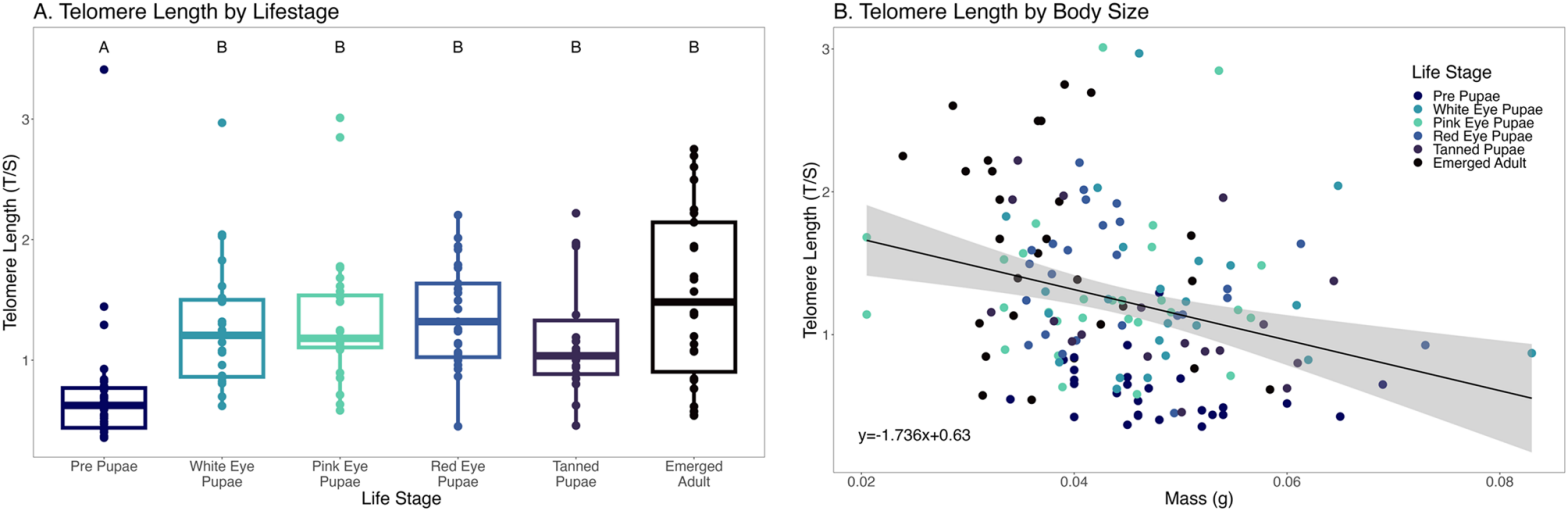
Telomere length in *M. rotundata*. (**A**) Telomere length across different developmental life stages. Letters represent significant differences between life stages from Tukey Comparison of Means. (**B**) Telomere length based on mass (g).

We measured telomere length across different developmental stages in *M. rotundata* to determine how telomere length changed throughout the lifespan, and across different masses. Life stage had a significant effect on telomere length (Table 2, Fig. 2A, Linear Model, F_5,132_ = 13.97; p < 0.0001). Telomeres were significantly shorter during the prepupal life stage, which is the earliest life stage tested in this experiment, than at subsequent life stages (Table 2). Mass significantly differed among life stages (Linear Model, F_5,143_ = 5.165, p < 0.001), and significantly predicted telomere length, (Fig. 2B, Linear Model, F_1,132_ = 5.55, p = 0.020). The interaction between mass and life stage was not significant (mass*life stage p = 0.590). Plate significantly influenced telomere length (F = 4.73, p = 0.001).

**Table 2:** Post-hoc comparisons of T/S ratios of different developmental stages in *M. rotundata.*

Life stage 1	Life stage 2	Estimate	Std error	z value	p value
White eye	Prepupae	0.744	0.149	4.987	< 0.001*
Pink Eye	Prepupae	0.741	0.137	5.422	< 0.001*
Red eye	Prepupae	0.752	0.138	5.451	< 0.001*
Tanning	Prepupae	0.633	0.155	4.089	0.001*
Adult	Prepupae	0.788	0.157	5.024	< 0.001*
Pink eye	White eye	− 0.003	0.146	− 0.021	1.000
Red eye	White eye	0.008	0.146	0.058	1.000
Tanning	White eye	− 0.111	0.165	− 0.674	0.984
Adult	White eye	0.045	0.166	0.268	0.999
Red eye	Pink eye	0.012	0.134	0.087	1.000
Tanning	Pink eye	− 0.108	0.151	− 0.717	0.979
Adult	Pink eye	0.048	0.153	0.312	0.999
Tanning	Red eye	− 0.119	0.154	− 0.779	0.970
Adult	Red eye	0.036	0.156	0.233	0.999
Adult	Tanning	0.156	0.170	0.916	0.941

Results from Multiple Comparisons of Linear Model test. *Significant difference.

### Osmia lignaria development

**Figure 3:**
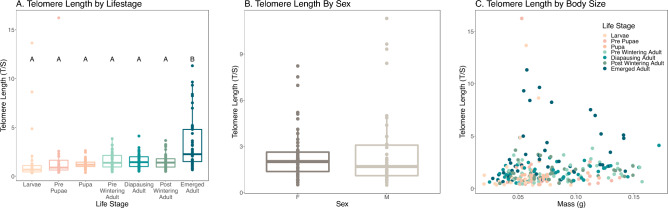
Telomere length in *O. lignaria*. (**A**) Telomere length across different developmental stages. Letters represent significant differences between life stages from Tukey Comparison of Means. **(B**) Comparison of telomere length between the sexes identified in the adult stage. (**C**) Telomere length based on mass (g).

We measured telomere length across different developmental stages in *O. lignaria* to determine how telomere length changes throughout the lifespan, and across different masses. Emerged bees had significantly longer telomeres than other developmental stages (Table 3, Fig. 3A, Linear Model, F_6, 263_ = 6.844; p < 0.001). Mass and sex were collected for adult bees in 2018, mass was collected for all life stages in 2021, neither mass nor sex significantly influenced telomere length, (Fig. 3B and C, Linear Model, mass F_1,231_ = 1.06, p = 0.304; sex F_1,95_ = 2.92 p = 0.747). There was also no significant interaction effect between mass and life stage on telomeres (Linear Model, mass*life stage F_6,231_ = 0.756 p = 0.605). Although, mass was significantly different between the different life stages (F_6,240_ = 3.545, p = 0.002) and males were smaller than females (Linear Model, F_1,99_ = 71.48, p < 0.001). Plate did not significantly influence telomere length (p = 0.061) Telomeres also varied by year/location (p = 0.008).

**Table 3:** Comparing T/S ratios of different developmental stages in *O. lignaria.*

Life stage 1	Life stage 2	Estimate	Std error	z value	p value
Prepupae	Larvae	0.046	0.458	0.100	1.000
Pupa	Larvae	− 0.457	0.441	− 1.036	0.944
Pre Winter	Larvae	− 0.156	0.434	− 0.359	0.999
Diapause	Larvae	− 0.182	0.432	− 0.421	0.999
Post Winter	Larvae	− 0.266	0.433	− 0.615	0.996
Emerged	Larvae	1.570	0.429	3.661	0.005*
Pupa	Prepupae	− 0.503	0.431	− 1.167	0.905
Pre Winter	Prepupae	− 0.201	0.423	− 0.476	0.999
Diapause	Prepupae	− 0.227	0.421	− 0.540	0.998
Post Winter	Prepupae	− 0.312	0.422	− 0.738	0.989
Emerged	Prepupae	1.525	0.419	3.640	0.005*
Pre Winter	Pupa	0.302	0.400	0.753	0.988
Diapause	Pupa	0.276	0.398	0.693	0.992
Post Winter	Pupa	0.191	0.398	0.479	0.999
Emerged	Pupa	2.028	0.394	5.146	< 0.001*
Diapause	Pre winter	− 0.026	0.387	− 0.067	1.000
Post Winter	Pre winter	− 0.111	0.387	− 0.286	0.999
Emerged	Pre winter	1.726	0.384	4.501	< 0.001*
Post Winter	Diapause	− 0.085	0.385	− 0.220	0.999
Emerged	Diapause	1.752	0.381	4.601	< 0.001*
Emerged	Post winter	1.837	0.381	4.822	< 0.001*

Results from multiple comparisons of linear model test. *significant difference.

### *Osmia lignaria* post-emergence adult lifespan

**Figure 4:**
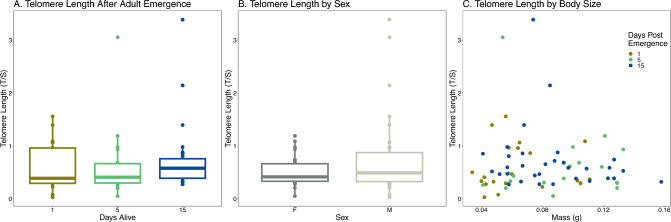
Telomere length in *O. lignaria* Adults. (**A**) Telomere length in adults by days post emergence. (**B**) Comparison of telomere length between the sexes. (**C**) Telomere length based on mass (g).

We measured telomere length in emerged adults to determine if telomere length changed in the adult lifespan after emergence in *O. lignaria*. Telomere length did not change throughout the post-emergence adult lifespan (Fig. 4A, Linear Model, F_2,62_ = 0.378, p = 0.687). Sex and mass did not affect telomere length in adults (Fig. 4B&C, Linear Model, Sex F_1,62_ = 3.426, p = 0.068; Mass F_1, 62_ = 1.043, p = 0.311). Mass was significantly different by day post emergence (Linear Model, F_2,69_ = 5.417, p = 0.007) and males are smaller than females (Linear Model, F_1,70_ = 759.88, p < 0.001) although this interaction did not significantly influence telomere length (F_2,62_ = 3.426, p = 0.898). Plate significantly influenced telomere length (F_1,62_ = 15.48 p < 0.001).

**Figure 5:**
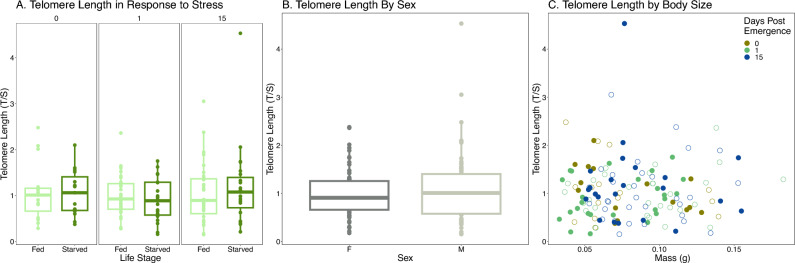
Telomere length in *O. lignaria* Adults in response to a nutritional stress. (**A**) Telomere length in adults by feeding treatment. (**B**) Telomere length between the sexes. (**C**) Telomere length based on mass (g), open circles represent bees in the fed treatment and closed circles represent bees in the starved treatment.

### *Osmia lignaria* adult feeding stress

We withheld food for 24 h to test whether feeding stress would impact telomere length 1 and 15 days after treatment. Telomere length did not differ in bees exposed to nutritional stress versus control (Fig. 5A, F_1,125_ = 0.017, p = 0.897). Consistent with our previous experiment, telomere length did not differ with respect to days post-emergence (F_2,125=_ 0.976, p = 0.380), sex (Fig. 5B, F_1,125_ = 0.896, p = 0.346), or mass (Fig. 5C, F_1,25_ = 0.002, p = 0.959). There was no effect of the interaction effect of feeding treatment and sex (F_2,136_ = 1.09, p = 0.298). There was no effect on the interaction of days post-emergence and feeding treatment on telomere length (F_2,125_ = 0.474, p = 0.623). Days alive did not have a significant impact on mass (F_2,136_ = 1.126, p = 0.327) but mass was different between the sexes with males being smaller than females (F_1,137_ = 159.57, p < 0.001). Plate had a significant influence on telomere length significant (F_6,125_ = 5.652, p < 0.001). We saw a decline in survival by day 6. Survival in the starved treatment by day 6 was 17%, and survival in the fed treatment was 31% by day 6.

## Discussion

In many organisms, telomere length declines with age^10,70–73^, is often negatively related to longevity^16–22^ and reflects early life conditions^8^. Interestingly, we report here that telomeres were *longer*, not shorter, in later developmental stages in both *M. rotundata* and *O. lignaria*. Telomeres were the shortest in the prepupal stage in *M. rotundata* and were longer during pupation and in adulthood. In the case of *O. lignaria,* telomeres were shorter in early developmental stages, including early adult stages, and became longer upon adult emergence. Thus, our study reveals that the pattern in two species of solitary bees differs from what is typically reported in vertebrates.

Interestingly, our study suggests that emergence from diapause, not life stage or age, is an important predictor for longer telomeres. Our results indicate that telomeres are longer when development resumes post-diapause. In our study, *M. rotundata* terminates diapause after the prepupal stage, correlating with longer telomeres in later developmental stages, and O. *lignaria* terminates diapause just before they emerge which correlated with longer telomeres in emerged adults. Periodic developmental arrest such as hibernation, diapause, or quiescence, has been shown to slow or reverse aging in many animal species^51^. Mammal hibernation and insect diapause share similar processes^44^. Hibernating mammals show increased telomere length post-hibernation^45^, which may be due to somatic maintenance that occurs during hibernation^46^. Similarly, insects that go through reproductive diapause have reduced signs of senescence^50^. The only previous study to date on telomeres and diapause in insects found that telomeres were shorter after diapause in queen bumblebees^37^ which is the opposite of the pattern we observed here. These contrasting patterns may be due to differences in diapause strategies and sociality between the species. Bumblebees are social bee species with a queen and workers. Koubová et al.^37^ measured telomere length in the fat bodies of queens, which are physiologically different than solitary bees. Caste and tissue-specific differences in senescence could explain the difference between the results and is worth pursuing in future studies. Longer telomeres in later life stages could be the result of selection bias, with individuals sampled at later timepoints being intrinsically longer lived and may have initially had longer telomeres. But, we do not think this was the case in this study because we didn’t see a wider range of variation in telomere length in earlier timepoints versus later timepoints. Instead, later life stages seen in Koubová et al.^37^ had broader distributions of telomere lengths in queens of different ages, which is not comparable to solitary bees. Our study is the first to demonstrate that emergence from diapause is associated with longer telomeres in later life stages and suggests that diapause may be a phase for cellular renewal in some species.

The absence of telomere shortening, and even lengthening in the case of post-diapausing individuals, suggests an active mechanism for maintaining telomeres in these bees. Upregulation of telomerase expression is a likely mechanism. Studies in social bees including honey bees and bumblebees indicate telomerase maintains telomere length throughout the lifespan and between castes^35–37,65^. Telomerase activity is often high during early growth and development. However, in eusocial bees, telomerase is upregulated in castes that are longer lived, not during development. Our study demonstrates that telomere length in solitary bees is maintained throughout diapause and over the adult lifespan, even when exposed to a stress treatment. This may indicate that telomeres, post-quiescence, are sufficiently long or are actively being maintained. While many studies in insects have characterized telomere structure^64,74,75^ a direct correlation between telomere length and longevity has not been determined^34–37,65^. One exception is the ant species *Lasius niger* in which longer-lived individuals have longer telomeres^73^. Telomerase expression in solitary bees is currently unknown but may be playing a role similarly to honeybees and bumblebees to maintain telomere length.

Our study also measured several factors previously reported to influence telomeres in some vertebrate studies including body size^76–78^, sex^79^, and nutritional stress^26^, but none of these influenced telomere dynamics across species in this study. Contrary to our predictions, nutritional stress experienced as an adult did not impact telomere length. In mammals, nutritional stress during rapid growth increases telomere shortening^71,72^ and it may be that nutritional stress experienced at earlier life-stages would also accelerate telomere loss in bees. In some cases, poor nutritional status is stressful^26^ and in others a reduction in nutrition has been shown to increase longevity^71^. We did not see a difference in telomere length based on one day of starvation after adult emergence. This may indicate that our treatment was not stressful enough. However, a short-term starvation period has been shown to reduce longevity in adult bees^56,80,81^. Our experiment attempted to balance providing a nutritional stress with maintaining survival to measure telomere length in the maximum longevity in captive bees. It may also be the case that solitary bees are resilient to nutritional stress at certain stages, or that any impacts on telomeres may have been delayed.

Female *O. lignaria* and *M. rotundata* are larger and have adult lifespans that are twice as long as males^58,59^. Because females are the longer-lived of the sexes, we expected telomere length to be longer in females, as has been demonstrated in ants^73^. However, telomere length was not significantly different between the sexes. Although the adult lifespan of females is longer than males, adult lifespan in general is a small proportion of the overall lifespan for solitary bees. For example, both sexes of *M. rotundata* spend approximately nine months in diapause^54^, making the extra week of the female adult lifespan a small fraction of the total annual lifespan. In vertebrates, the sexes do not have differences in telomere length even if there are differences in lifespan^79^. We also tested for an association between telomere length and mass. Larger animals tend to live longer when compared between species, but larger individuals within a species tend to have shorter lifespans^77,78,82,83^. However, there was no relationship between mass and telomere length. We only saw this pattern in *M. rotundata*, with smaller individuals having longer telomeres. There may be no differences in telomere length based on sex or size because the timing of telomere restoration in these bees may occur right before adult emergence, after size and sex have been determined. Body size in solitary bees is largely determined by the amount of food the mother provides the larvae^58,84–90^. Bees that are larger as adults were provided and consumed more food as larvae. Most of the life stages in this study were after the larval feeding period, and bees do not consume food during diapause. We did not find an effect of mass across both species on telomere length, even when accounting for life stage. Although this study was cross-sectional, this result suggests that individuals who eat more as larvae do not invest those additional resources into telomere length disproportionately more than smaller individuals.

While it is clear that telomeres are playing a role in senescence in many organisms, it may not be representative of cellular aging and could play a different role in solitary bees. We see an increase in telomere length at different life stages in the two species, which appear to be more closely correlated to emergence from diapause than developmental stage. More research is needed to determine to what extent telomere length is indicative of ageing in insects. Insect populations exhibit the characteristics of senescence as evidenced by a comprehensive long-term study^91^, but this has not been tied to cellular markers of aging. Our study establishes that shorter telomeres during development are longer when development resumes as demonstrated by increases in telomere length in both bee species after exposure to warm temperatures, triggering emergence from diapause. Telomere length was maintained in the adult stage in *O. lignaria* which may indicate that this potential cellular renewal is maintained in adult stages. Our results show that telomere length does not decline with chronological age, but lengthening may occur following diapause. Future studies should focus on how telomerase expression changes throughout diapause in solitary bees which may give insights to when bees are prioritizing cellular repair.

### Supplementary Information

Supplementary Information 1.

Supplementary Information 2.

## Data Availability

All data generated or analyzed during this study are included in this published article [and its supplementary information files].
