# Supplementary material for: Telomere length is longer following diapause in two solitary bee species
Source: Sci Rep. 2024 May 16;14:11208. doi: 10.1038/s41598-024-61613-2 (PMC11099051; doi:10.1038/s41598-024-61613-2)
Supplement: Supplementary file 2 — Supplementary Information 2. [file 41598_2024_61613_MOESM2_ESM.docx]

Telomere length is longer following diapause in two solitary bee species

Courtney Grula et al.

Supplementary materials

Below are results from a multiple comparisons linear model test using the *lme4* package using plate as a random effect in the model.

**(Random effect) *Megachile rotundata* Development**. We measured telomere length across different developmental stages in *M. rotundata* to determine how telomere length changed throughout the lifespan, and across different weights. Life stage had a significant effect on telomere length (Table 2, Fig. 2A, Linear Model, F_5,143_= 9.05; p < 0.0001). Telomeres were significantly shorter during the prepupal life stage, which is the earliest life stage tested in this experiment, than at subsequent life stages (Table 2). Mass significantly differed among life stages (Linear Model, F_5,143_=5.165, p <0.001), and significantly predicted telomere length, (Fig. 2B, Linear Model, F_1,143_=5.19, p = 0.024) The interaction between mass and life stage was not significant (mass*life stage p= 0.535).

| Life stage 1 | Life stage 2 | Estimate | Std error | z value | Pvlaue |
| --- | --- | --- | --- | --- | --- |
| White eye | Prepupae | 0.728061 | 0.148670 | 4.897 | < 1e-04 |
| Pink Eye | Prepupae | 0.735929 | 0.136379 | 5.396 | < 1e-04 |
| Red eye | Prepupae | 0.756771 | 0.137897 | 5.488 | < 1e-04 |
| Tanning | Prepupae | 0.634163 | 0.154568 | 4.103 | 0.000572 |
| Adult | Prepupae | 0.790440 | 0.156519 | 5.050 | < 1e-04 |
| Pink eye | White eye | 0.007868 | 0.145239 | 0.054 | 1.000000 |
| Red eye | White eye | 0.028710 | 0.146073 | 0.197 | 0.999959 |
| Tanning | White eye | -0.093898 | 0.163860 | -0.573 | 0.992703 |
| Adult | White eye | 0.062379 | 0.165238 | 0.378 | 0.998997 |
| Red eye | Pink eye | 0.020842 | 0.133739 | 0.156 | 0.999987 |
| Tanning | Pink eye | -0.101767 | 0.150581 | -0.676 | 0.984473 |
| Adult | Pink eye | 0.054510 | 0.152440 | 0.358 | 0.999229 |
| Tanning | Red eye | -0.122608 | 0.153194 | -0.800 | 0.967229 |
| Adult | Red eye | 0.033669 | 0.154322 | 0.218 | 0.999932 |
| Adult | Tanning | 0.156277 | 0.169535 | 0.922 | 0.940550 |

Results from Multiple Comparisons of Linear Model test. *significant difference

**(Random effect) *Osmia lignaria* Development.** We measured telomere length across different developmental stages in *O. lignaria* to determine how telomere length changes throughout the lifespan, and across different weights. Emerged bees had significantly longer telomeres than other developmental stages (Table 3, Fig. 3A, Linear Model, F_6, 272_ = 6.68; p < 0.001). Mass and sex were only collected only for adult bees in 2018, but mass was collected for all life stages in 2021, mass nor sex significantly influenced telomere length, (Fig. 3B&C, Linear Model, mass F_1,247_ = 0.125, p= 0.724; sex F_1,101_ = 0.911 p= 0.342). There was also no significant interaction effect between mass and life stage on telomeres (Linear Model, mass*life stage F_6,247_ =0.661, p=0.681). Mass did significantly different between the different life stages (F_6,240_ = 3.545, p= 0.002) and sex with males being smaller than females (Linear Model, F_1,99_ = 71.48, p <0.001). Telomeres also varied by year/location (p=0.015).

| Life stage | Life stage 2 | Estimate | Std error | z value | Pvlaue |
| --- | --- | --- | --- | --- | --- |
| Prepupae | Larvae | -0.0576427 | 0.4552475 | -0.127 | 1.00000 |
| Pupa | Larvae | -0.5017464 | 0.4368755 | -1.148 | 0.91243 |
| Pre Winter | Larvae | -0.2220279 | 0.4304635 | -0.516 | 0.99863 |
| Diapause | Larvae | -0.2215533 | 0.4295189 | -0.516 | 0.99863 |
| Post Winter | Larvae | -0.3375955 | 0.4294708 | -0.786 | 0.98626 |
| Emerged | Larvae | 1.5450642 | 0.4261012 | 3.626 | 0.00527 ** |
| Pupa | Prepupae | -0.4441037 | 0.4255063 | -1.044 | 0.94347 |
| Pre Winter | Prepupae | -0.1643852 | 0.4170284 | -0.394 | 0.99971 |
| Diapause | Prepupae | 0.1639106 | 0.4155410 | -0.394 | 0.99971 |
| Post Winter | Prepupae | -0.2799528 | 0.4172917 | -0.671 | 0.99410 |
| Emerged | Prepupae | 1.6027069 | 0.4132008 | 3.879 | 0.00210 ** |
| Pre Winter | Pupa | 0.2797185 | 0.3957380 | 0.707 | 0.99219 |
| Diapause | Pupa | 0.2801931 | 0.3949630 | 0.709 | 0.99203 |
| Post Winter | Pupa | 0.1641508 | 0.3952366 | 0.415 | 0.99960 |
| Emerged | Pupa | 2.0468106 | 0.3911416 | 5.233 | < 0.001 ** |
| Diapause | Pre winter | 0.0004745 | 0.3823483 | 0.001 | 1.00000 |
| Post Winter | Pre winter | -0.1155677 | 0.3825130 | -0.302 | 0.99994 |
| Emerged | Pre winter | 1.7670921 | 0.3784333 | 4.669 | < 0.001 *** |
| Post Winter | Diapause | -0.1160422 | 0.3804503 | -0.305 | 0.99993 |
| Emerged | Diapause | 1.7666175 | 0.3758528 | 4.700 | < 0.001 *** |
| Emerged | Post winter | 1.8826597 | 0.3765658 | 5.000 | < 0.001 *** |

(**Random effect) *Osmia lignaria* Post-Emergence Adult Lifespan** We measured telomere length in emerged adults to determine if telomere length changed in the adult lifespan after emergence in *O. lignaria*. Telomere length did not change throughout the post-emergence adult lifespan (Fig. 4A, Linear Model, F_2,70_=0.060, p = 0.941). Mass did not affect telomere length in adults (Fig. 4B&CMass F_1, 70_=1.834, p= 0.181). Mass was significantly different by day post emergence (Linear Model, F_2,69_= 5.417, p= 0.007) although this interaction did not significantly influence telomeres (F_2,70_=0.113, p=0.893) and, males are smaller than females (Linear Model, F_1,70_ = 759.88, p <0.001). Telomere length was significantly different by sex (Linear Model, Sex F_1,62_= 3.425, p= 0.069).

**(Random effect) *Osmia lignaria* Adult Feeding Stress.** We withheld food for 24 hours to test whether feeding stress would impact telomere length 1 and 15 days after treatment. Telomere length did not differ in bees exposed to nutritional stress versus control (Fig 5A, F_1,125_= 0.001, p=0.974). In corroboration of our previous experiment, telomere length did not differ with respect to days post-emergence (F_2, 125=_ 0.576, p = 0.564), or mass (Fig. 5C, F_1,25_=0.142, p=0.707). Telomere length was not impacted by sex (Fig 5B, F_1,125_= 0.897, p=0.345). There was no impact on the interaction between feeding treatment and sex on telomere length (F_1,125_=0.857, p=0.774) of There was no impact on the interaction of days post-emergence and feeding treatment on telomere length (F_2,125_=0.443, p =0.643). Days alive did not have a significant impact on mass (F_2,136_=1.126, p=0.327) but mass was different between the sexes with males being smaller than females (F_1,137_=159.57, p <0.001). We saw a decline in survival by day 6. Survival in the starved treatment by day 6 was 17%, and survival in the fed treatment was 31% by day 6.
